# Clinical and Capillaroscopic Modifications of the Psoriatic Plaque during Therapy: Observations with Oral Acitretin

**DOI:** 10.1155/2013/781942

**Published:** 2013-09-23

**Authors:** Giuseppe Stinco, Cinzia Buligan, Enzo Errichetti, Francesca Valent, Pasquale Patrone

**Affiliations:** ^1^Department of Experimental and Clinical Medicine,Institute of Dermatology, University of Udine, Ospedale “San Michele” di Gemona, Piazza Rodolone 1, Gemona del Friuli, 33013 Udine, Italy; ^2^Department of Medical and Biological Sciences, Institute of Hygiene and Clinical Epidemiology, University of Udine, via Colugna 50, 33100 Udine, Italy

## Abstract

Psoriasis is considered to be an inflammatory autoimmune disease, where angiogenesis plays an undefined pathogenetic role. The well-known changes of the superficial microvasculature in the psoriatic plaque can be easily assessed in vivo by videocapillaroscopy. In the last years, several studies reported the clinical and capillaroscopic response of the psoriatic plaque during different topical and systemic treatments. In the present work we evaluated the effects of acitretin (0.8 mg/kg/day) on videocapillaroscopic alterations and the clinical response in 11 patients affected by plaque psoriasis at the baseline (T0) and after 4 (T1), 8 (T2), and 12 (T3) weeks. A clinical improvement during the treatment with a complete clinical healing of the plaque in 7 of the 11 patients was observed. The typical “basket-weave” capillaries of the psoriatic lesions showed a reduction of 65.4% in diameter at the end of the study; only 3 patients returned to a normal capillaroscopic pattern. As observed during previous our studies, we found a discrepancy between clinical and capillaroscopic results, with a far greater improvement in the first than in the second. This finding could be in agreement with a secondary role of blood vessels in the pathogenesis and persistence of psoriatic lesions.

## 1. Introduction

Psoriasis is a chronic debilitating inflammatory dermatosis characterized by recurring well-demarcated reddish skin plaques covered with silvery scales. The histological hallmarks observed within psoriatic lesions include a thickened epidermis from rapid keratinocyte proliferation and aberrant differentiation, a reduced or absent granular layer, an increase of vascularity in the papillary dermis, and dense clusters of inflammatory cells composed of T cells and dendritic cells in the dermis and CD8+ T cells and neutrophils in the epidermis [1]. Among these changes, vascular alterations are thought to be the first to occur, thus supporting a fundamental role of angiogenesis and microangiopathy in the development of psoriasis through the promotion of an inflammatory response [1–3]. 

Several angiogenic mediators are involved in psoriasis including vascular endothelial growth factor (VEGF), hypoxia-inducible factors, angiopoietins, and proangiogenic cytokines, such as tumour necrosis factor (TNF), interleukin- (IL-) 8, and IL-17 [2]. Following the release of these molecules, the papillary dermal microvessels in psoriatic lesions show both functional changes (increased expression of inflammation-associated adhesion molecules such as E-selectin, intercellular adhesion molecule-1, and vascular cell-adhesion molecule-1) and morphological modifications (prominent elongation, tortuosity, and dilatation of capillaries) [1, 2]. These last alterations can be easily assessed noninvasively in vivo by videocapillaroscopy [2]. 

The capillaroscopic pattern of psoriatic lesions is characteristic and consists of dilated, elongated, and convoluted capillaries with a “bushy” or “basket-weave” appearance arranged regularly, parallel to the cutaneous surface. These capillaries appear as dotted, globular, or coiled (glomerular) vessels on dermoscopy (at ×10 magnification). The diameter of the vessels typically appears increased in the lesional areas compared to the 5-6 *μ*m of the capillaries in the healthy skin, which show a typical reticulate network aspect [4, 5]. 

In the last years the modifications of the psoriatic microcirculation have been monitored during the use of several drugs, and, in particular, our research group have studied the clinical and capillaroscopic response of the psoriatic plaque during both topical and systemic treatments [3–10]. A discrepancy between clinical and microcirculatory improvement was always observed. Here we report the effects of acitretin on videocapillaroscopic alterations and clinical response in psoriatic patients.

## 2. Materials and Methods

Eleven consecutive patients suffering from moderate to severe psoriasis with a psoriasis area and severity index (PASI) of at least 12 points measured during the first examination were enrolled. All the patients were treated with acitretin at the dose of 0.8 mg/kg/die for at least 12 weeks. Main exclusion criteria were pregnancy or breastfeeding, heart disease or severe liver diseases, kidney disorders, tumours, ongoing systemic infections, significant abnormalities or laboratory investigations, treatment by other systemic therapies for psoriasis or phototherapy during the last 2 months, and treatment by topical therapies, such as corticosteroids and vitamin D analogue preparations during the last 2 weeks. Only treatments already in course for other preexisting pathologies were allowed and not discontinued. The study consisted in both a clinical and videocapillaroscopic examination of a selected psoriatic plaque. For each patient, the largest psoriatic plaque on the right arm was selected, and its centre was identified by characterizing precise anatomical benchmarks and measuring the distance from three bony prominences. From each capillaroscopic image of the centre of the plaque, the diameter of 3 different “baskets” was measured and the mean was then calculated and taken as the patient's reference value. The clinical assessment of the chosen plaque was performed by assigning a score between 0 and 4 for erythema, infiltration, and scaling (0 = none, 1 = minimal, 2 = mild, 3 = moderate, and 4 = severe). The severity ratings for each of these three parameters were then added to give the plaque severity score (range 0–12). Four clinical and capillaroscopic examinations were planned: inclusion examination (T0) and three follow-up examinations at week 4 (T1), week 8 (T2), and week 12 (T3). To standardize the technique, the examinations were always performed by the same investigator. To minimize possible observer bias, every image was recorded and then analysed afterward by the same operator under blinded conditions. The consistency of this double measurement resulted in almost perfect reproducibility scores. Full medical examinations were carried out at each time point, and the laboratory parameters were recorded to monitor the acitretin treatment adequately. A contact digital capillaroscope (Dermascope; Medici Medical SRL, Castelfranco Emilia, Modena, Italy) was used for videocapillaroscopic imaging, with a 300x lens. Each patient was acclimatized for 30 minutes in a temperature-controlled room at 24 ± 1°C. Each area examined was painted lightly with lemon oil to improve contrast by reducing reflective glare and increasing skin translucency. The capillaroscope was composed of a central body with a 100 W cold halogen lamp fitted with a manual luminous intensity control device, a probe consisting of a 2 m long flexible cable enclosing an optical fibre bundle, a video signal-processing unit, and a connection cable between the signal-processing unit and the optical terminal. The optical terminal comprised a colour microcamera and a support to hold the various 300x magnification lenses used [4–7].

All procedures followed were in accordance with the ethical standards of the responsible committee on human experimentation (institutional and national) and with the Helsinki Declaration of 1975, as revised in 2000 and 2008. The study protocol was approved by the internal review board of the Department of Experimental and Clinical Medicine at the University of Udine. A written consent for the participation in the study was obtained from all the patients.

All values are expressed as mean ± SD. The statistical significance of the microcirculatory reduction and clinical improvement was calculated by means of Student's *t*-test. *P* < 0.05 was considered significant. 

## 3. Results 

The eleven patients, 4 women and 7 men, presented with a mean age of 41 years (range 39–65), and, at baseline, the mean PASI score was 13.7 ± 3.1. The capillaroscopic evaluation of the centre of the selected plaque demonstrated in all the patients a typical psoriatic pattern with capillary loops that assume a basket-weave aspect.

**Figure 1:**
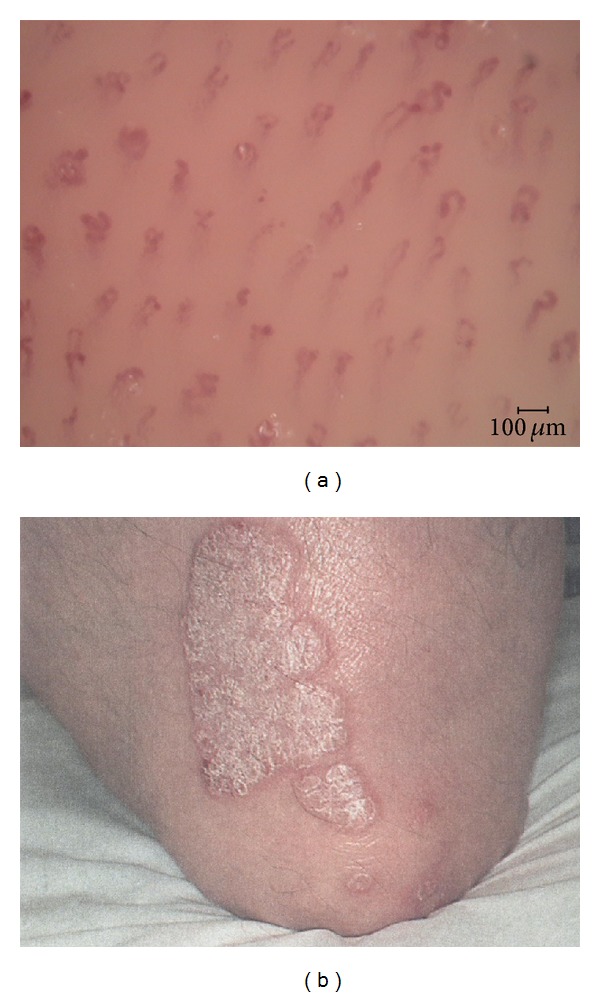
Capillaroscopic image (a) of the centre of the psoriatic plaque (b) at T0.

The initial clinical evaluation (T0) of the plaque showed a mean score of 7.12 ± 0.50, while the mean diameter of capillaries was 70.02 ± 10.60 *μ*m (Figure 1). At subsequent evaluations, the clinical improvement in erythema, infiltration, and scaling was evidenced with a score of 4.08 ± 0.62 at T1 (*P* < 0.0001) and 1.83 ± 0.45 at T2 (*P* < 0.0001). Also the “basket” diameter decreased from a mean value of 47.69 ± 7.22 *μ*m at T1 (*P* < 0.0001) to 32.26 ± 8.33 *μ*m at T2 (*P* = 0.0002).

**Figure 2:**
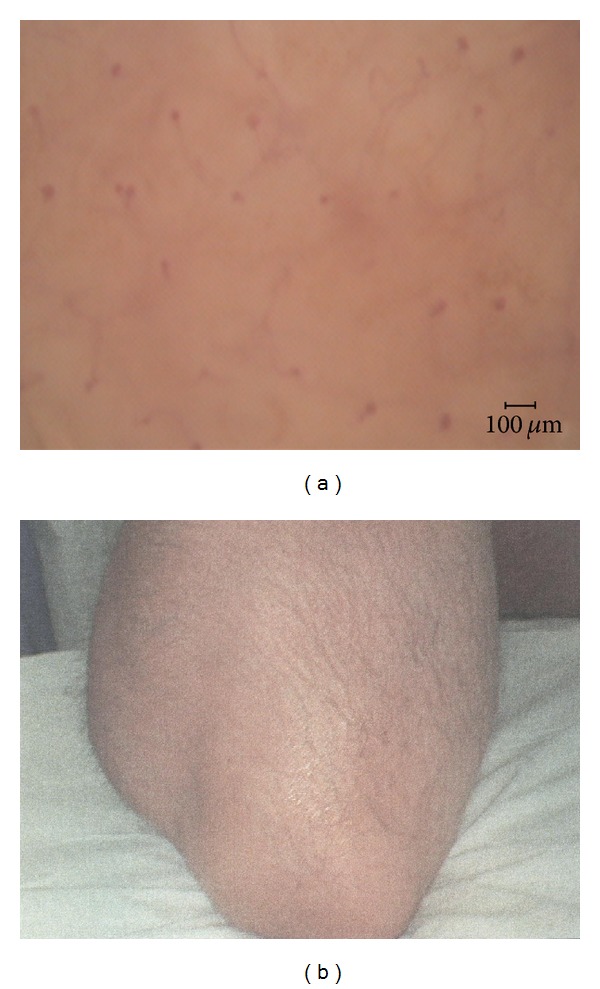
Capillaroscopic image (a) of the centre of the resolved psoriatic plaque (b) at T3 in the same patient of Figure 1.

After 12 weeks of treatment (T3), 7 of the 11 patients had a complete clinical resolution of the psoriatic plaque. In the remaining 4 patients, the mean clinical score was 0.85 ± 0.65 (*P* = 0.0008). Only in 3 patients the capillaroscopic pattern returned to normality, while in 8 patients the capillaroscopic evaluation showed a score of 24.23 ± 5.03 *μ*m (*P* = 0.0146) (Figure 2).

The results are reported in Table 1.

## 4. Discussion 

**Figure 3:**
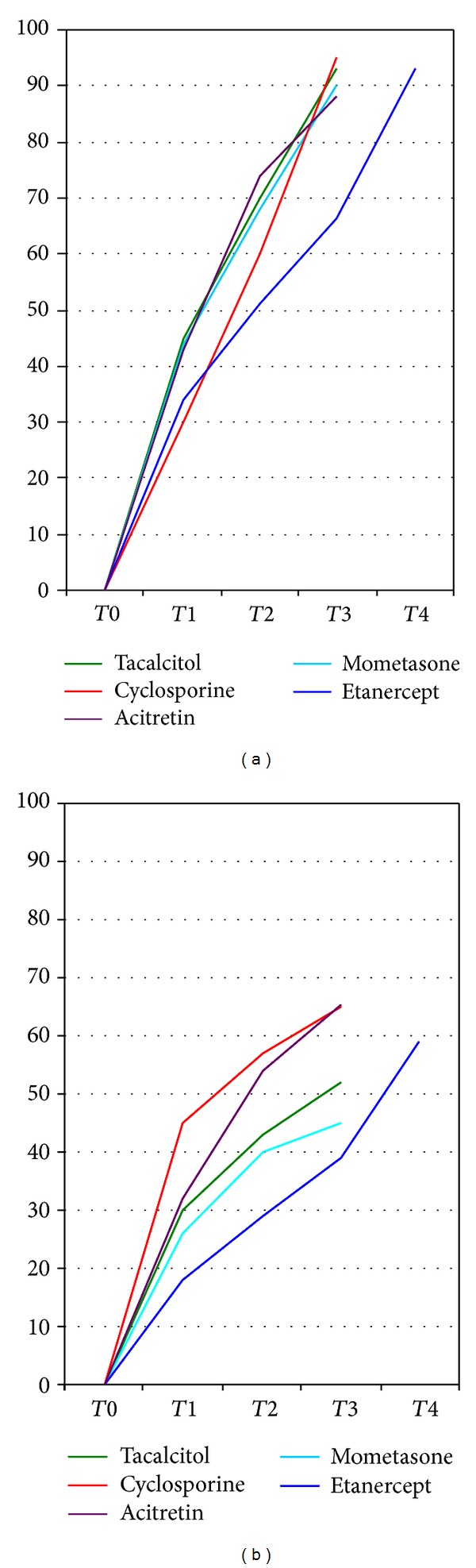
Mean improvement in clinical score (a) and mean reduction in basket diameter (b) at the psoriatic plaque treated with tacalcitol, mometasone furoate, cyclosporine A, etanercept [4–7], and acitretin at each time point as a percentage of baseline values. T0: start of treatment; T1: after 4 weeks; T2: after 8 weeks; T3: after 12 weeks; T4 (only for etanercept [5]): after 24 weeks.

The first study monitoring the capillaroscopic modifications during treatment with antipsoriatic drugs dates back to 1994, when Strumia et al. reported a reduction in the length and tortuosity of the capillary loops of psoriatic lesions in five patients after 6 weeks of therapy with topical tacalcitol [8]. Subsequently, Rosina et al. evaluated the effects of topical calcipotriol and betamethasone, alone and in combination, on the typical “bushy” capillaries of psoriatic lesions, reporting that after only 15 days of therapy, the “bushy” diameter and capillary number showed a marked reduction, which was significantly higher with calcipotriol and betamethasone association compared with the single therapies. Furthermore, the authors underlined that capillaroscopic modifications were more rapid than clinical improvement, emphasizing an important role of microvascular alterations in the maintenance of psoriatic lesions [9]. With regard to systemic therapies, De Angelis et al. observed that a single infusion of infliximab induced a rapid improvement in clinical features and significant changes in the morphology of the capillary loops in psoriatic plaques, which appeared less tortuous and dilated with an evident reduction in shape and size. The number of “bushy” loops was also reduced, with complete regression after the second infusion of the drug [10]. Similar results were reported by Campanati et al. who observed a significant decrease in the total number of tortuous and enlarged capillaries after 12 weeks of therapy with etanercept. The authors reported that the improvement of capillaroscopic changes resulted significantly related with both the clinical improvement and the local decrease in the immunohistochemical expression of VEGF, thus interpreting the vascular alterations as primary pathogenetic promoters of cutaneous manifestations of psoriasis [3]. This hypothesis would be supported by two main data: the onset of dermal microangiopathy in the course of psoriasis usually anticipates a clinical relapse, and a treatment promoting a microangiopathy regression leads to a more stable clinical healing, with a relapse of psoriatic lesions more likely if vascular alterations persist [3, 5]. The simultaneous clinical and capillaroscopic improvement reported by Campanati et al. using etanercept at a dose of 50 mg twice per week [3] have not been observed in our recent study on psoriatic patients treated with etanercept at a dose of 50 mg once per week [5]; these different findings might be explained, at least in part, by the different dosage used. Moreover, the results reported by Campanati et al. are also in contrast with data observed in the present study as well as with our prior works evaluating the capillaroscopic modifications during treatment with other topical and systemic antipsoriatic drugs, including topical tacalcitol [4], mometasone furoate [7], and cyclosporin A [6]. In general, albeit in all cases, we have found a significant improvement of capillaroscopic alterations of psoriatic plaques with a decrease of diameter of the “bushy” capillaries after 12 weeks of therapy, and we have noted a constant discrepancy between the clinical and capillaroscopic results, with a far greater improvement in the first than in the second [4–7] (Figure 3). This finding could be in agreement with a secondary role of blood vessels in the pathogenesis and persistence of psoriatic lesions, with some extravascular factors that would allow persistence of the capillaroscopic alterations even in the presence of minimal clinical disease, as emphasized by Hern and Mortimer [11]. This interpretation is valued by the fact that psoriatic recurrence not only is restricted to areas where an altered capillaroscopic pattern persists at the site of previous lesions, but also occurs in areas without such a pattern [5, 11].

Interestingly, neither our previous studies nor the other works have found a real normalization of the capillaroscopic pattern of the lesions to the typical reticulate network aspect, even after remission of the psoriatic plaques, except for some of our patients treated with topical tacalcitol and mometasone furoate, respectively, about 33% and 17% of subjects who had clinically healed after 12 weeks of therapy [4, 7]. In the present work we have experienced a capillaroscopic normalization only in 3 patients (27%) with a clinical remission (Figures 1 and 2). The reason for the normalization of the microvascular alterations only in few cases remains obscure. Anyhow, the frequent persistence of videocapillaroscopic disturbances in clinically healed psoriatic plaques might represent an indication that these lesions are not completely healed but only partially remitted to a subclinical stage. Consequently, it is possible that these lesions may recur more easily than those who were healed also videocapillaroscopically. However, this point still remains unresolved given that no study has assessed how long it takes a drug to obtain capillaroscopic healing of a plaque and whether return to normal capillaroscopic aspect is actually correlated with the duration of remission. The lack of long-term studies of this kind could be explained, at least in part, by some limitation in the use of videocapillaroscopy. Particularly, since it requires specific equipment, the technique is demanding in terms of costs and time, and it is not an easily applied method in daily practice. This raises the question whether more easily and widely applied diagnostic tools, such as dermoscopy, may play such a role in the future.

## 5. Conclusion

In the last years the capillaroscopic changes of psoriatic lesions have been monitored during the use of several drugs with conflicting results. Some authors have reported a correlation between clinical and capillaroscopic improvements, thus interpreting the vascular alterations as possible primary pathogenetic promoters of psoriatic manifestations. Instead, in the present study as well as in our other works, we found a constant discrepancy between clinical and capillaroscopic results, with a far greater improvement in the first than in the second, thus supporting a putative role of blood vessels. Therefore, although it is well known that microvascular changes are of fundamental importance in development of psoriasis, the debate on their primary or secondary role still remains open. Anyhow, their frequent persistence in resolved psoriatic lesions assessed by videocapillaroscopy raises questions about whether psoriatic plaques that do not have a normal capillaroscopic pattern can ever be considered “healed” or only partially remitted to a subclinical stage. In this last view, videocapillaroscopy could be even more valuable, since it would allow a more accurate estimation of the treatment response, comparing to naked-eye examination alone. Further studies are needed to clarify the above-mentioned questions. 

## Figures and Tables

**Table 1:** Diameters of the “baskets” and clinical score following treatment with acitretin.

Time from start of study	Clinical evaluation Clinical score Mean ± SD	Capillaroscopic evaluation “Basket” diameter (*μ*m) Mean ± SD
T0—Baseline	7.12 ± 0.50	70.02 ± 10.60
T1—4 weeks	4.08 ± 0.62	47.69 ± 7.22
T2—8 weeks	1.83 ± 0.45	32.26 ± 8.33

T3—12 weeks	0.85 ± 0.65	24.23 ± 5.03
